# Lipoprotein subfraction patterns throughout gestation in The Gambia: changes in subfraction composition and their relationships with infant birth weights

**DOI:** 10.1186/s12944-023-01776-5

**Published:** 2023-02-03

**Authors:** Jessica G. Woo, John T. Melchior, Debi K. Swertfeger, Alan T. Remaley, Ebrima A. Sise, Fatou Sosseh, Jeffrey A. Welge, Andrew M. Prentice, W. Sean Davidson, Sophie E. Moore, Laura A. Woollett

**Affiliations:** 1 Departments of Pediatrics and Environmental and Public Health Sciences, University of Cincinnati College of Medicine, Cincinnati, OH USA; 2 Division of Biostatistics and Epidemiology, Cincinnati Children’s Hospital Medical Center, Cincinnati, OH USA; 3 Department of Pathology and Laboratory Medicine, University of Cincinnati College of Medicine, Cincinnati, OH USA; 4 Biological Sciences Division, Pacific Northwest National Laboratory, WA Richland, USA; 5 Division of Biomedical Informatics, Cincinnati Children’s Hospital Medical Center, Cincinnati, OH USA; 6 Lipoprotein Metabolism Section, Cardio-Pulmonary Branch, National Heart, Lung, and Blood Institute, National Institutes of Health, Bethesda, MD USA; 7 MRC Unit The Gambia, London School of Hygiene and Tropical Medicine, Banjul, The Gambia; 8 Department of Psychiatry and Behavioral Neuroscience, University of Cincinnati College of Medicine, Cincinnati, OH USA; 9 Department of Women and Children’s Health, King’s College London, London, UK

**Keywords:** High-density lipoprotein, Low-density lipoprotein, Triglyceride-rich lipoprotein, Proteomics, Subspecies

## Abstract

**Background:**

Lipoprotein subfraction concentrations have been shown to change as gestation progresses in resource-rich settings. The objective of the current study was to evaluate the impact of pregnancy on different-sized lipoprotein particle concentrations and compositions in a resource-poor setting.

**Method:**

Samples were collected from pregnant women in rural Gambia at enrollment (8–20 weeks), 20 weeks, and 30 weeks of gestation. Concentrations of different-sized high-density, low-density, and triglyceride-rich lipoprotein particles (HDL, LDL, and TRL, respectively) were measured by nuclear magnetic resonance in 126 pooled plasma samples from a subset of women. HDL was isolated and the HDL proteome evaluated using mass spectroscopy. Subfraction concentrations from women in The Gambia were also compared to concentrations in women in the U.S. in mid gestation.

**Results:**

Total lipoprotein particles and all-sized TRL, LDL, and HDL particle concentrations increased during gestation, with the exception of medium-sized LDL and HDL particles which decreased. Subfraction concentrations were not associated with infant birth weights, though relationships were found between some lipoprotein subfraction concentrations in women with normal versus low birth weight infants (< 2500 kg). HDL’s proteome also changed during gestation, showing enrichment in proteins associated with metal ion binding, hemostasis, lipid metabolism, protease inhibitors, proteolysis, and complement activation. Compared to women in the U.S., Gambian women had lower large- and small-sized LDL and HDL concentrations, but similar medium-sized LDL and HDL concentrations.

**Conclusions:**

Most lipoprotein subfraction concentrations increase throughout pregnancy in Gambian women and are lower in Gambian vs U.S. women, the exception being medium-sized LDL and HDL particle concentrations which decrease during gestation and are similar in both cohorts of women. The proteomes of HDL also change in ways to support gestation. These changes warrant further study to determine how a lack of change or different changes could impact negative pregnancy outcomes.

**Supplementary Information:**

The online version contains supplementary material available at 10.1186/s12944-023-01776-5.

## Introduction

Metabolic changes occurring during pregnancy are required for healthy development of the infant. These include 50–100% increases in maternal triglyceride and cholesterol concentrations [1–3], which are likely fuelled by fetal demands [4, 5]. The circulating lipids are distributed into soluble lipid-protein complexes called lipoproteins. Triglycerides are carried primarily as triglyceride-rich lipoproteins (TRL) which include intestinally-derived chylomicrons and liver-derived very-low density lipoproteins (VLDL) while cholesterol is primarily carried as low-density lipoproteins (LDL) and high-density lipoproteins (HDL). Increases in lipid concentrations observed in pregnancy are typically associated with greater increases in LDL-cholesterol (LDL-C) and TRL-triglyceride (TRL-TG) concentrations with modest to no increases in HDL-C concentrations [1–3].

The focus on lipoprotein metabolism during pregnancy typically centers around the lipid transport pathway as lipoproteins are best-known as vehicles that deliver fats for fuel and for cell membrane biogenesis. However, recent research has demonstrated that lipoproteins are much more complex and can associate with a large array of proteins that impart unexpected functions such as inflammation, immunity, hemostasis/protease inhibition, and cell/heparin binding, in addition to lipid transport and metabolism [6, 7]. Many of these studies have shown direct associations between maternal triglyceride and/or LDL-C concentrations with fetal growth rates [8, 9]. In contrast, results with HDL-C are mixed, most likely due to only measuring the cholesterol carried by HDL which makes up a small percentage of HDL mass [10].

Of the major lipoproteins, HDL exhibits the most compositional and functional diversity. A plethora of biophysical studies that fractionate HDL by size [11, 12] or proteins that sit on the surface [13, 14] has led to the notion that HDL is comprised of a spectrum of distinct particles with unique protein complements that govern particle function [7, 15–17]. The different HDL subfractions (different-sized particles) [16] appear to shift in concert with metabolic perturbations in individuals with diabetes and obesity [18–21]. Recently, HDL was speciated using size exclusion chromatography and demonstrated a striking change in particle size distributions and HDL proteome in a small cohort of pregnant women in Cincinnati, Ohio, U.S. [22].

In contrast, the apolipoprotein B (APOB)-containing lipoproteins are less diverse in composition, though they differ in size. These lipoproteins are often associated with lipid transport and are the primary lipoproteins measured in pregnancy-related studies due to their role in what has been known as a key function in pregnancy, that being the requirement of lipids by the fetal tissues. As with HDL, pregnancy affects the distribution of these APOB-containing particles, as shown previously [22–25].

Thus, the goal of the current study was to build on previous observations and evaluate lipoprotein subfraction concentrations and the HDL proteomes in pregnant women of a resource-poor setting, rural Gambia, where there is a greater risk of adverse outcomes. Relationships of LDL and HDL subfractions with birth weights were also assessed based on past studies from this cohort [3]. Finally, lipoprotein subfraction concentrations of the Gambian women were compared to concentrations of women at a comparable state of gestation in a more resource-rich setting in the U.S. [22] to help delineate potential causes of adverse outcomes in resource-poor settings because a majority of the world’s low birth weight (LBW) infants are born in resource-poor settings [26]. This is among the first studies analyzing lipoprotein subfraction concentrations and HDL proteomes longitudinally in pregnant women of a resource-poor setting.

## Materials and methods

### Subjects

The Early Nutrition and Immune Development trial (ENID; ISRCTN49285450) was a randomised, partially blinded trial of antenatal and infant nutritional supplementation conducted in the rural West Kiang region of The Gambia as described previously [3, 27]. The current study is a secondary analyses of samples collected from women enrolled in ENID. Plasma was pooled from sets of three women to obtain enough sample volume for the analyses. Pooling requirements were based on initial BMI of the women, gestational age at enrollment, infant birth weight, and gestational age at birth; based on the lack of effect of interventions on plasma lipids [3], the interventions were not included in the criteria to pool samples. Out of a total of 800 women with live births [3], and excluding women with gestational ages < 37.9 weeks and women whose infants were missing birth weights or gestational age at birth, 126 pooled samples were generated at each time point; not all samples were used as not all samples could be matched with other samples based on pooling criteria. Only women with infants in quintile 1 (Q1, lowest birth weight infants), Q3 (middle birth weight infants), and Q5 (highest birth weight infants) were analyzed for a total of 42 samples per quintile at each gestational age.

Briefly, blood samples were collected from women at the MRC Keneba clinic at enrollment (8–20 weeks of gestation; median 13.5 weeks) and at 20 and 30 weeks of gestation after an overnight fast. Whole blood samples were processed, and plasma samples were frozen at -70 °C until analyzed. The ENID trial and this sub-study were approved by the joint Gambian Government/Medical Research Council (MRC) Unit and additional approval was obtained from the Institutional Review Board at the University of Cincinnati as described [3]. The original trial followed Good Clinical Practice Standards and the current version of the Helsinki Declaration.

### Lipids, lipoprotein subfractions, and apolipoprotein concentrations

Nuclear magnetic resonance (NMR; Vantera® Clinical Analyzer) spectroscopy was used to measure total lipid, lipoprotein-lipid and apolipoprotein concentrations, and the concentrations of different-sized lipoprotein particles (TLR, LDL, and HDL subfractions) in pooled plasma samples using the LP4 algorithm as previously described [22, 28].

### HDL proteome

The HDL proteome was measured in plasma in which APOB-containing lipoproteins (TRL and LDL) were removed by precipitation using heparin and magnesium chloride [22]; 28 of the 42 pooled samples in each quintile were used for these analyses for a total of 84 samples at each time point. Lipid-bound proteins were isolated by incubating APOB-depleted plasma with a lipid-removal agent (LRA; calcium silicate hydrate) [29]. The lipid-bound proteins were washed, and proteins were digested directly off the LRA with sequencing-grade trypsin. Digested peptides were reduced, alkylated, dried under vacuum and stored at -20 ºC until analysis by liquid chromatography-mass spectrometry. Peptides were separated from one another on a reverse phase column (ACQUITY UPLC C18, Waters) prior to introduction onto the mass spectrometer (6550 Q-TOF, Agilent). Peptides were identified and quantitated using spectral counts [30]; raw counts for each sample were summed and presented as a percent of the total counts. Identified proteins and peptides were constrained to 99.9% and 95.0%, respectively, and protein inclusion required identification of at least 3 unique peptides.

### Gene ontology analyses

Using gene ontology (GO) analyses associated with Scaffold (Proteome Software, Inc., Portland, OR 97,219, U.S.), proteins were grouped based on functions; GO is often used to analyze aspects of a gene product’s biological function. Functional groups included metal ion binding, hemostasis, protease inhibitors, proteolysis, lipid metabolism, acute inflammatory response, and complements. Protein percentages were grouped in the various pathways, summed, and averages obtained.

### Statistical analysis

Descriptive statistics were calculated and compared across the primary grouping variable, birth weight quintile using chi-squared analysis or Fisher’s exact test for categorical variables and analysis of variance (ANOVA) for continuous variables. Post-hoc pairwise comparisons using unpaired t-tests among groups were conducted if the omnibus tests were significant (*P* < 0.05). Longitudinal analysis of lipids and lipoprotein concentrations and sizes across pregnancy timepoints was conducted using generalized linear mixed models, which account for correlations within person over time, and permits each individual her own intercept. Covariates of interest included maternal pre-pregnancy body mass index (BMI), gestational age at enrollment, pregnancy visit (enrollment, 20 weeks’ or 30 weeks’ gestation), gestational age at delivery and birth weight quintile (Q1, Q3 or Q5). The primary comparisons were between visits during pregnancy, evaluated using differences of adjusted means, with *P* < 0.05 considered significant. Each of the lipoprotein subfractions was modelled separately, with values below the limit of detection analyzed as 0 s. The HDL proteomic data were analyzed using linear mixed models across the three visits during pregnancy, adjusting for maternal pre-pregnancy BMI, infant birth weight, and infant gestational age at delivery. Applying Bonferroni correction across 82 tests, *P* values < 0.006 were considered significant. The summed proteins used for the GO analyses were compared by one-way ANOVA, followed by Holm-Sidak analyses.

Secondary analyses were also conducted to evaluate lipoprotein subfractions by birth weight quintile or by LBW status (< 2500 g vs ≥ 2500 g). Birth weight quintiles were analyzed for all lipoprotein subfractions, whereas only LDL and HDL subfractions were analyzed for associations by LBW based on results from past analyses of the ENID samples [3]. Analyses were conducted longitudinally for birth weight quintile differences, and cross-sectionally for both birth weight quintiles and LBW comparisons. Of note, comparisons were not made between women with term and preterm infants due to the low number of preterm births in this cohort [3]. Differences in values at enrollment were also assessed after grouping gestational age at sampling above or below the median age of 13.5 weeks’ gestation.

Samples from Gambian women taken at 20 weeks’ gestation were also compared cross-sectionally with mid-pregnancy (18–24 week) samples from women in Cincinnati, Ohio [22]; samples from both cohorts were analyzed using the same instrument and by the same laboratory. These comparisons were conducted using generalized linear regression separately for each lipoprotein species, adjusting for maternal pre-pregnancy BMI, which was lower in women of The Gambia.

## Results

### Study population

The clinical characteristics of the subset of women used in this study are shown in Table S1. There was no significant difference in the BMI between the women with different sized infants nor in gestational age at enrollment. The median gestational age at birth of the smallest (Q1) and middle-sized (Q3) infants were ≈1 week earlier than the largest (Q5) infants. Infant birth weights were significantly different between women in Q1, Q3, and Q5 by design.

### Longitudinal lipoprotein-lipid, total lipid, and subfraction concentrations

**Fig. 1:**
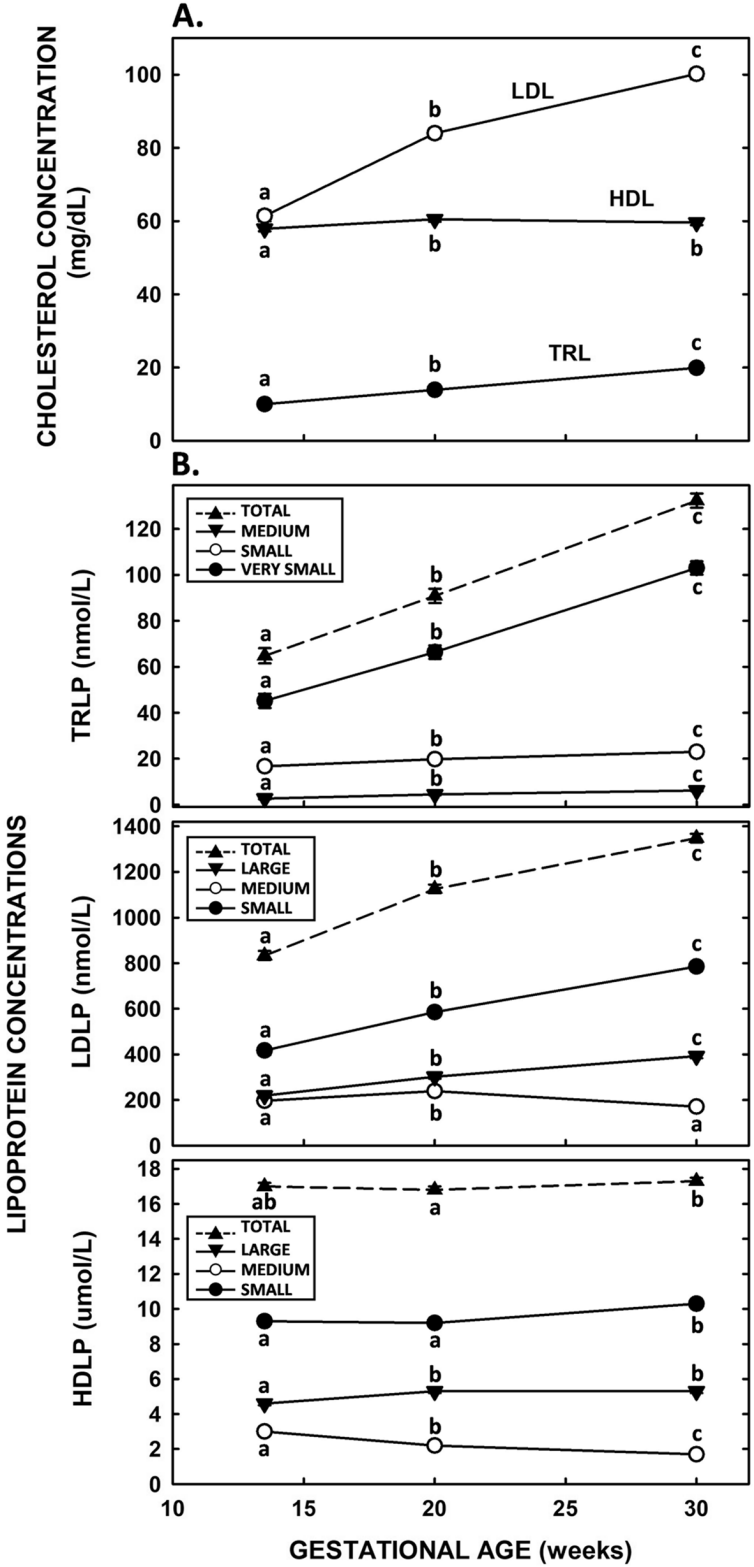
Longitudinal lipid and lipoprotein subfraction concentrations in pregnant women of The Gambia. Data in all figures represent adjusted means ± SEM for all pooled samples (sample numbers = 101 at enrollment, 125 at 20 weeks, and 125 at 30 weeks). Different letters represent differences between gestational ages of each lipoprotein. **A** Lipoprotein-cholesterol concentrations at enrollment (mean 13.5 weeks of gestation), and 20 and 30 weeks of gestation. Lipoprotein-cholesterol, measured by NMR, is presented as triglyceride-rich lipoproteins (TRL) (closed circles), low-density lipoproteins (LDL) (open circles), and high-density lipoproteins (HDL) (closed triangles). **B** Lipoprotein subfraction concentrations at enrollment, 20 weeks and 30 weeks of gestation. Top panel. Total TRLP concentrations are presented as up triangles with a dashed line, medium TRLP as down triangles, small TRLP as open circles, and very small as closed circles. Middle panel. Total LDLP concentrations are presented as up triangle with a dashed line, large LDLP as down triangles, medium LDLP as open circles, and small LDLP as closed circles. Bottom panel. Total HDLP concentrations are presented as up triangle with a dashed line, large HDLP as down triangles, medium HDLP as open circles, and small HDLP as closed circles

Similar to enzymatic analyses [3], cholesterol and triglyceride concentrations measured by NMR increased as gestation progressed (*P* < 0.0001; Table S2). For lipoprotein-C concentrations (Fig. 1A), TRL-C concentrations almost doubled and LDL-C concentrations increased about 60% (*P* < 0.0001 for both), whereas HDL-C concentrations minimally increased (*P* = 0.02). The changes in concentrations of APOB (Table S2), the main apolipoprotein carried by LDL and TRL, paralleled LDL-C and TRL-C concentrations. Concentrations of APOA1, the main apolipoprotein on HDL, increased about 25% (Table S2) even though HDL-C concentrations changed minimally.

For lipoprotein subfractions, the concentration of total TRLP almost doubled from enrollment to 30 weeks of gestation (Fig. 1B, top panel), due to a more than doubling of the very small particles and slight increases in the small- and medium-sized particles (*P* < 0.0001 for all). Total LDLP concentrations increased throughout gestation (Fig. 1B, middle panel), with the small and large LDLP having the greatest increases (*P* < 0.0001 for both). Medium-sized particles increased (*P* = 0.03) and then decreased (*P* = 0.0004) as gestation progressed. The minimal increase in HDLP concentrations (*P* < 0.007) (Fig. 1B, bottom panel) were associated with a redistribution of HDL particle sizes; both large- and small-sized HDL particles increased, whereas medium-sized particles decreased (*P* < 0.0001 for all). This redistribution of different-sized particles was associated with an increase in the average size of HDL (9.95 ± 0.21 vs 10.09 ± 0.02 nm; *P* < 0.0001).

When the lipoprotein subfraction levels and lipids in women who entered the study at gestational ages less than and greater than the mean gestational age at enrollment (13.5 weeks) were compared, similar patterns were found as those detected throughout gestation. Of APOB lipoproteins, very small-sized TRLP and large-sized LDLP concentrations were greater at the older gestational age (*P* < 0.001 and 0.005, respectively). Medium-sized HDLP concentrations decreased (*P* < 0.0001) and large HDLP concentrations increased (*P* < 0.0002) as gestation increased from < 13.5 to > 13.5 weeks.

### Associations of lipoprotein subfractions and apolipoproteins with infant birth weight

Surprisingly, none of the TRLP, LDLP or HDLP subfractions differed between the women with Q1, Q3, or Q5 infants, adjusting for visit, maternal BMI, gestational age at enrollment, and gestational age at birth. To expand on previous results showing relationships between HDL-C or LDL-C and LBW or small-for-gestational-age infants [3], sensitivity analyses were performed that evaluated differences in HDL and LDL subfractions, and APOA1 and APOB concentrations between women giving birth to LBW infants compared to normal birth weight (NBW) infants. At enrollment, women with LBW infants had lower concentrations of APOA1 (*P* = 0.03) and a trend for lower HDLP concentrations (*P* = 0.066; Table S3) vs women with NBW infants. At 20 weeks’ gestation, there were higher medium-sized HDLP concentrations (*P* = 0.017) and a trend for lower large-sized HDLP concentrations (*P* = 0.051) in the women with LBW infants. At 30 weeks’ gestation, the women with LBW infants had lower concentrations of small-sized LDLP (*P* = 0.03) and a trend for lower total LDLP concentrations (*P* = 0.069). After adjusting for BMI, gestational age at birth, and gestational age at enrollment, only the small LDLP concentrations at 30 weeks remained significantly different between the two groups (*P* = 0.02).

### HDL proteome and GO analyses

**Fig. 2:**
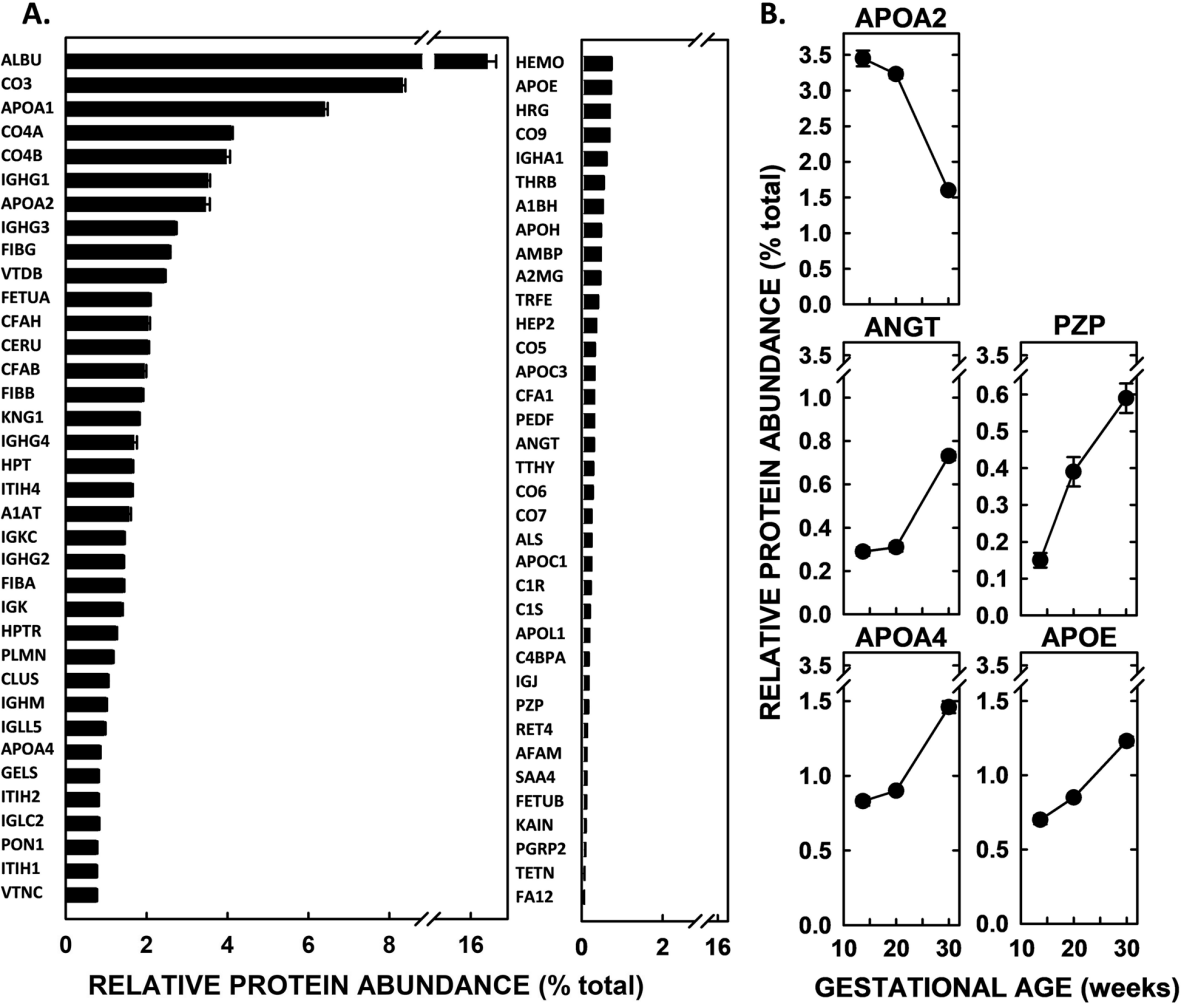
HDL proteome at enrollment of women in The Gambia. HDL was isolated by APOB-depletion, and proteins were analyzed by LC–MS and abundance quantified using spectral counts as percent of total spectral counts. Data represent means (*n* = 84) ± SEM. **A** Spectral counts of all proteins detected, shown in descending order of abundance. **B** The five proteins shown with the largest changes in relative abundance of proteins (> 50%) from enrollment to 30 weeks of gestation

The proteome analysis of HDL at enrollment identified 72 proteins. The ten most prevalent proteins were apolipoproteins (APOA1 and APOA2), complement factors (CO3, CO4A, and CO4B), and proteins associated with immune function (CO3, CO4A, CO4B, IGHG1, and IGHG3), clotting (FIBG), and lipid binding (APOA1, APOA2, VTDB) (Fig. 2A). More proteins were detected on HDL at both 20 (78 proteins) and 30 (86 proteins) weeks’ gestation. The relative abundance of many proteins changed between the gestational ages (Table S4), with APOA2, ANGT, PZP, APOA4, and APOE changing by the greatest percentage (Fig. 2B). Twenty-two proteins were not present at enrollment but were found at 20 and 30 weeks’ gestation, including pregnancy-specific glycoproteins, complement factors, and sex-related hormones.

**Fig. 3:**
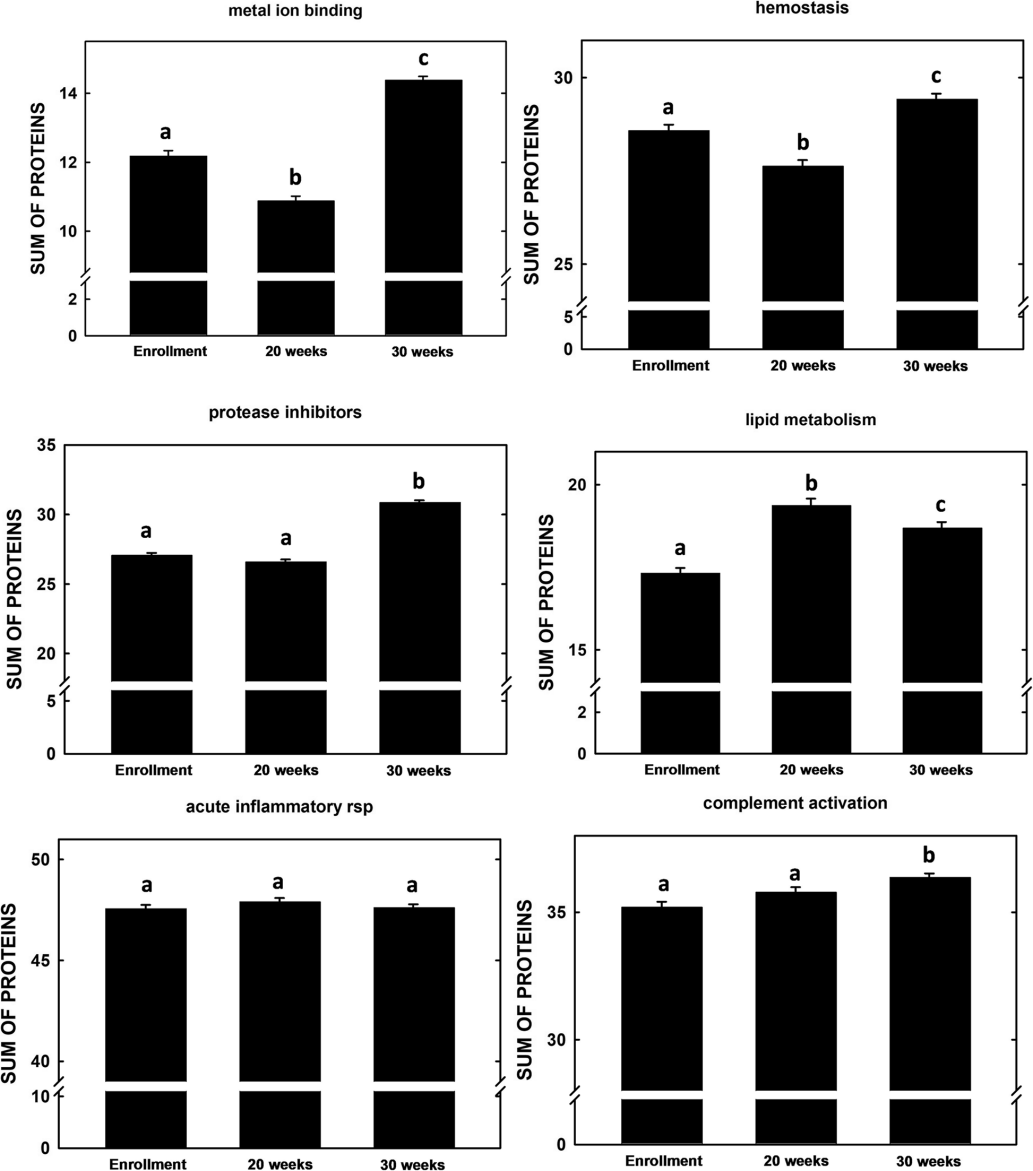
Summation of relative amounts of each protein in GO-defined pathways in women in The Gambia. Albumin was not included in the analyses as it is often a contaminant of this method. Data represent means (*n* = 84) ± SEM

Proteins were grouped by function (Table S5) and summed (Fig. 3). Most of the pathways showed an increase in the relative abundance of proteins associated with each specific pathway by 30 weeks’ gestation, except for proteins associated with the acute inflammatory response which remained similar throughout gestation; metal ion binding, hemostasis, protease inhibitors, and lipid metabolism, lipid metabolism (all *P* < 0.00005) and complement activation (*P* = 0.0017), and acute inflammatory response (*P* = 0.37).

### Comparison of lipoprotein particle concentrations between The Gambia and Cincinnati

**Fig. 4:**
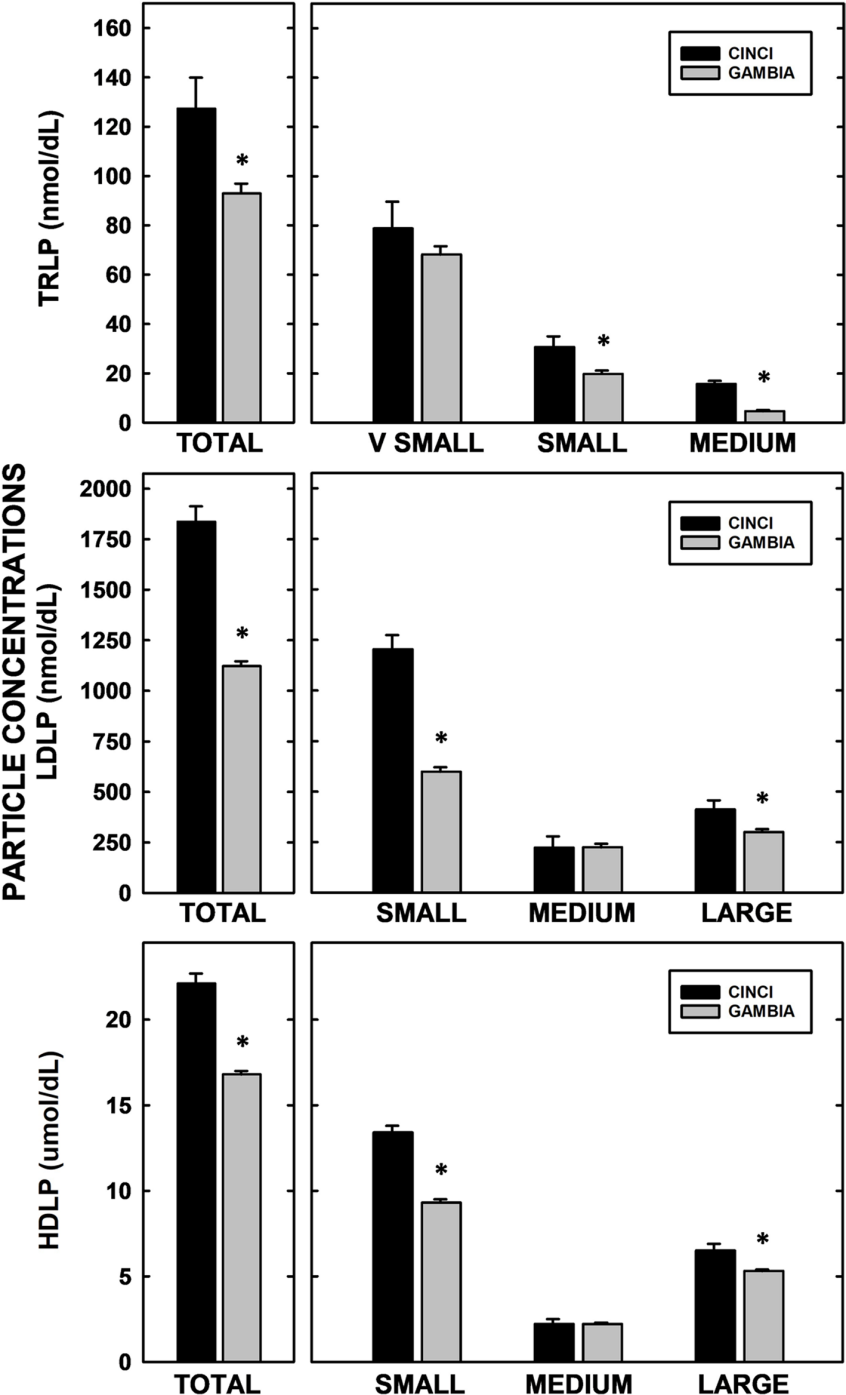
Comparisons of lipoprotein particle concentrations in pregnant women of resource-rich and resource-poor areas. Lipoprotein particle concentrations (TRL-top panel, LDL-middle panel, HDL-bottom panel) were measured in women of The Gambia (gray) and Cincinnati (black). Samples were collected at 20 weeks (The Gambia) or 18–24 weeks (Cincinnati) of gestation. Total lipoprotein concentrations are shown on the left and different-sized species on the right. Data represent adjusted means ± SEM for samples (The Gambia, *n* = 125; Cincinnati, *n* = 19). Different letters represent differences between women from the two cohorts between total particle and different-sized species concentrations

As it is uncommon to analyze lipoprotein subfraction concentrations in a resource-poor setting, lipoprotein subfractions of Gambian women were compared to those of women in a more resource-rich setting from Cincinnati, Ohio, U.S. of similar maternal and gestational ages [3, 14]. Triglyceride, cholesterol, TRL-TG, TRL-C, LDL-C, HDL-C, APOB, and APOA1 concentrations were lower in Gambian vs U.S. women (Table 1), as reported previously [3]. Likewise, total TRLP, LDLP, and HDLP concentrations were lower in women of The Gambia vs women in Cincinnati (Fig. 4) (*P* = 0.015, < 0.0001, and < 0.0001, respectively), and Gambian women also had lower concentrations of small-sized LDL and HDL particles (*P* < 0.0001 for both) and large-sized LDL and HDL particles (*P* = 0.03 and 0.001, respectively). In contrast, and unexpectantly, medium-sized LDLP and HDLP concentrations were similar in both cohorts. For TRLP, small- and medium-sized particle concentrations were lower in women of The Gambia (*P* = 0.026 and < 0.0001, respectively), and very small particle concentrations were similar with women in Cincinnati. BMI was largely unrelated to lipoprotein subfractions and concentration, even though the BMI was greater in Cincinnati women.

**Table 1:** Lipids and apolipoproteins in pregnant^a^ women from resource-poor and resource-rich areas

	The Gambia	Cincinnati	*p value*
Triglyceride (mg/dL)	41.5 ± 1.4	92.5 ± 4.4	< *0.0001*
Cholesterol (mg/dL)	157.5 ± 2.5	232.8 ± 7.9	< *0.0001*
TRL-TG (mg/dL)	25.9 ± 1.3	64.0 ± 4.2	< *0.0001*
TRL-C (mg/dL)	14.2 ± 0.6	21.7 ± 1.9	*0.0004*
LDL-C (mg/dL)	83.3 ± 2.0	138.7 ± 6.4	< *0.0001*
HDL-C (mg/dL)	60.1 ± 0.8	72.4 ± 2.6	< *0.0001*
APOA1 (mg/dL)	135.3 ± 1.8	173.9 ± 5.7	< *0.0001*
APOB (mg/dL)	63.0 ± 1.4	101.5 ± 4.3	< *0.0001*

^a^Women were 20 weeks of gestation (The Gambia) and 18–24 weeks of gestation (Cincinnati)

Values represent means ± SEM, adjusting for maternal BMI

## Discussion

The goal of the current study was to examine the impact of pregnancy on different-sized lipoprotein particle concentrations and compositions throughout gestation in a resource-poor setting. These lipoprotein subfractions have not frequently been studied, mostly because lipoproteins were until recently thought to be related only to lipid transport. Women in a resource-poor setting were the focus of these studies as women in these settings are at an increased risk for adverse outcomes, including low birthweight and preterm birth [26, 31], with the thought that the current studies will define normal changes which should occur in gestation of uncomplicated pregnancies and might help define causes of adverse outcomes. Likewise, unique differences in particle concentrations between women in resource-poor and a more resource-rich setting could provide insights into the increased risk for adverse outcomes in women of resource-poor settings.

Changes in HDL should theoretically have the biggest impact on pregnancy outcomes since these particles carry the greatest variety of proteins [7, 15–21] which impact a number of functions involved in pregnancy [32]. It has been found that there is a redistribution of HDL subfractions during pregnancy in resource-rich settings, with an increase in the large-sized HDL and a decrease in the medium-sized HDL in mid and late gestation [22, 33]. These changes are not unexpected as estrogen increases activity of cholesteryl ester transfer protein and decreases hepatic lipase activity, leading to a redistribution of lipids and an increase in the larger particles [33–37]. As estrogen drives this effect and is needed to maintain pregnancy, it is not surprising that the Gambian women also had a redistribution to more large and less medium HDLP as gestation progressed. However, since both large and small HDLP concentrations were lower in the Gambian women, but medium-sized HDLP concentrations were similar, there was ≈30% more of the medium-sized HDL in the Gambian women vs the U.S. women (13.3% vs 9.9%, respectively). Interestingly, when the 10 most prevalent HDL proteins previously described in the Cincinnati cohort [22] were compared to the 10 most prevalent HDL proteins in the Gambian samples collected at 20 weeks’ gestation, 4 unique proteins were detected in each cohort (Table S6). The Cincinnati samples had proteins associated with hemostasis and the Gambian samples had proteins associated with immunity, suggesting functionally different HDL.

The question remains—what are the functions of these medium-sized HDLP and are they beneficial or detrimental to pregnancy? Though the functions of these particles are not fully known, data suggest that the particles may be involved with poorer pregnancy outcomes. For example, medium-sized HDLP concentrations were greater in Gambian women with LBW infants at 20 weeks’ gestation, though the differences were not significant once adjusted for gestational age. Likewise, another study measured higher medium-sized HDL concentrations in mid-gestation in women with preterm infants [38]. These results, combined with the fact that APOA1 and HDL-C concentrations are lower in women with LBW infants [3], suggest that measurements of different-sized HDL species could be a useful biomarker for LBW and could help identify novel mechanisms leading to LBW or preterm infants. It is also possible that the relationships between different-sized lipoprotein species with adverse outcomes, for example LBW and preterm births, will differ between resource-rich and resource-poor settings. There has been substantial variability in the reported relationships of HDLP and HDL-C concentrations and adverse pregnancy outcomes, including LBW and preterm births, which could be due partly to the study location and if it was performed in a resource-rich or resource-poor setting [39]. However, this study in a resource-poor setting and a prior study in a resource-rich setting both showed no relationship between NBW and lipoprotein subfractions [40], so it is reasonable to speculate that differences in lipoprotein subfractions may be unique to adverse outcomes.

As a start in understanding whether and how HDL is involved in gestation, the HDL proteome was examined throughout gestation. It was found that the proteins with the greatest increases across pregnancy were involved in pathways associated with in the maintenance of pregnancy, including activation of the complement cascade, inflammation and immunity, and regulation of blood pressure. Two of the proteins with the greatest increase throughout pregnancy were APOA4 and APOE, both proteins which have anti-inflammatory properties. These results may suggest a role of HDL in controlling inflammation as numerous cells of the placenta synthesize and secrete pro-inflammatory cytokines throughout pregnancy [32]. One protein, APOA2 was reduced > 50% during gestation, which also suggests a shift in HDL function as HDL-APOA2 mediates cholesterol efflux [14]; interestingly APOA2 is carried on medium-sized HDL [22].

When proteins being used as abundance indicators in various pathways related to pregnancy were summed, the proteins increased as gestation progressed, except for the acute inflammatory response (no change). These pathways are all involved in maintaining pregnancy and examples include: 1) ensuring adequate sources of micronutrients/metals in tissues, 2) establishing appropriate innate immunity activity via complement activation, 3) maintaining hemostatic balance changes to ensure coagulation during parturition, and 4) transporting lipids between tissues [41–44]. While analyses like this can give an idea of metabolic changes over time, it is important to note that such summations can be heavily influenced by large changes in one relatively abundant protein. For example, though many proteins in the lipid metabolism pathway increased during gestation, as seen at 20 and 30 weeks of gestation, there was a decrease in activity between 20 and 30 weeks, which was primarily driven by the dramatic decrease in APOA2 from 20 to 30 weeks.

As with large and small HDLP concentrations, there was an increase in the APOB-containing particle concentrations in this resource-poor setting as found in resource-rich settings. These increases could be due to fetal demands since TRLP are likely the main source of energy-producing lipids for the placenta and fetus and LDLP carry cholesterol which is required for membrane formation. An increase in TRLP formation and secretion are consistent with the higher estrogen levels found during pregnancy as estrogens increase production of VLDL [45], which is often associated with an increase in small LDL [24]. As with HDL, not all sizes of LDL increased in the Gambian women; small- and large-sized LDL concentrations increased whereas medium-sized LDL concentrations did not increase. When levels of APOB lipoproteins in resource-richer and resource-poorer women were compared, there was a greater proportion of medium-sized LDL in the Gambian women (20.0% vs 12.1%), which is parallel with what occurred with HDL. Interestingly, the relationship of LDLP and TRLP and infant birthweight appears to differ in resource-rich and resource-poor settings as TRLP concentrations were not related to birthweight, including LBW, in The Gambia but higher TRL-TG levels were related to increased birthweights in cohorts of resource-rich settings [40].

## Strengths and limitations

A major strength is that this study benefits from a standardized collection of samples that were taken longitudinally throughout pregnancy from women in a resource-poor setting, that being The Gambia. Novel markers that were not previously studied in resource-poor settings were analyzed. The concentrations of the novel markers were also compared to concentrations in resource-rich settings collected at a similar time of gestation. Limitations also occurred. Due to sample volume constraints, samples had to be pooled, resulting in average levels that could potentially mask relationships between individual-level clinical characteristics and lipoprotein profiles. Also, while maternal BMI was not associated with any outcomes assessed, other unmeasured characteristics or characteristics that were not included in the pooling criteria, including maternal age, may play a role in lipoprotein concentration or particle size during gestation.

## Conclusion

Lipoprotein subfraction concentrations change longitudinally in pregnant Gambian women, with increases of the small- and large-sized HDLP and LDLP concentrations and decreases in medium-sized HDLP and LDLP concentrations. The changes in HDL subfraction concentrations are accompanied with changes in the HDL proteome and potentially with HDL functionality, and include proteins and pathways (assessed by GO analyses) which support pregnancy. Though the function of all lipoprotein subfractions are not known, the long-term goal of these measurements is to be able to use concentrations of specific-sized lipoprotein particles or lipoprotein-associated proteins to identify women at risk of adverse outcomes, such as medium-sized HDLP or HDL proteins involved in specific pathways. Results can also be used to identify pathways that may be involved adverse outcomes that were not originally known, such as possibly APOA2 levels and cholesterol efflux. It must be considered that different-sized lipoprotein particles will be related to adverse outcomes in settings with different resources.

## Supplementary Information

**Additional file 1: Table S1.** Clinical characteristics of pregnant women in The Gambia. **Table S2.** Longitudinal concentrations of lipids and apolipoproteins of pregnant women in The Gambia. **Table S3.** Lipids and apolipoproteins in pregnant women from The Gambia with NBW* or LBW* infants. **Table S4.** Relative amounts of proteins in APOB depleted plasma during gestation. **Supplemental Table 5.** GO Pathway Proteins. **Supplemental Table 6.** Top proteins in HDL of pregnant women.

## Data Availability

There are no datasets or materials to share.

## Abbreviations

TRL: Triglyceride-rich lipoproteins
VLDL: Very low-density lipoproteins
HDL: High-density lipoproteins
LDL: Low-density lipoproteins
LDL-C: Low-density lipoprotein-cholesterol
TRL-TG: TRL triglyceride
APO: Apolipoprotein
LBW: Low birth weight
ENID: Early Nutrition and Immune Development
Q1: Quintile 1
MRC: Medical Research Council
LRA: Lipid-removal agent
GO: Gene ontology
BMI: Basal metabolic rate
