# Supplementary material for: Lipoprotein subfraction patterns throughout gestation in The Gambia: changes in subfraction composition and their relationships with infant birth weights
Source: Lipids Health Dis. 2023 Feb 3;22:19. doi: 10.1186/s12944-023-01776-5 (PMC9896684; doi:10.1186/s12944-023-01776-5)
Supplement: Supplementary file 1 — Additional file 1: Table S1. Clinical characteristics of pregnant women in The Gambia. Table S2. Longitudinal concentrations of lipids and apolipoproteins of pregnant women in The Gambia. Table S3. Lipids and apolipoproteins in pregnant women from The Gambia with NBW* or LBW* infants. Table S4. Relative amounts of proteins in APOB depleted plasma during gestation. Supplemental Table 5. GO Pathway Proteins. Supplemental Table 6. Top proteins in HDL of pregnant women. [file 12944_2023_1776_MOESM1_ESM.docx]

Table S1. Clinical characteristics of pregnant women in The Gambia

Q1* Q3* Q5* Total *P value*

BMI 20.5±0.3 21.3±0.5 21.8±0.5 21.2±0.3 *0.125*

GA (weeks, enrollment)^Ψ^ 14.1±0.5 13.3±0.5 14.0±0.5 13.8±0.3 *0.459*

GA (weeks, birth) 39.8±0.2^a^ 40.2±0.2^a^  40.9±0.2^b^ 40.3±0.1 *<0.001*

Birthweight (kg) 2.50±0.02^a^  2.99±0.01^b^  3.60±0.03^c^  3.03±0.04 *<0.001*

*Q1, Q3, Q5 – birth weight quintiles.

^Ψ^ GA-gestational age. Enrollment occurred from 8-20 weeks of gestation.

Values represent unadjusted means ± SEM. Different letters represent differences (*P*<0.05)

between quintiles.

Table S2. Longitudinal concentrations of lipids and apolipoproteins

of pregnant women in The Gambia

Enrollment* 20 weeks 30 weeks *P value*

Triglyceride (mg/dL) 31.5±1.0^a^ 41.2±0.9^b^ 52.7±0.9^c^ *<0.0001*

Cholesterol (mg/dL) 129.3±2.0^a^ 158.4±1.9^b^ 179.7±1.9^c^ *<0.0001*

TRL-TG (md/dL) 19.1±0.9^a^ 25.5±0.8^b^ 35.0±0.8^c^ *<0.0001*

APOA1 (mg/dL) 130.7±1.5^a^ 136.0±1.5^b^ 140.5±1.5^c^ *<0.0001*

APOB (mg/dL) 46.6±1.1^a^ 63.2±1.1^b^ 76.9±1.1^c^ *<0.0001*

*Enrollment occurred from 8-20 weeks of gestation.

Values represent means ± SEM, adjusted for birthweight quintile, maternal BMI, gestational

age at enrolment, and gestational age at birth. Different letters represent differences

(*P*<0.05) between gestational ages.

Table S3. Lipids and apolipoproteins in pregnant women from The Gambia with NBW* or LBW* infants

NBW LBW *P value*

Enrollment

LDLP 847±22 794±30 *0.16*

LLDLP 217.5±9.3 237.8±17.1 *0.35*

MLDLP 198.6±14.2 192.3±31.2 *0.85*

SLDLP 430.4±17.8 363.8±30.0 *0.11*

APOB 47.1±1.3 45.0±1.6 *0.30*

HDLP 17.2±0.2 16.3±0.4 *0.066*

LHDLP 4.69±0.10 4.38±0.25 *0.24*

MHDLP 3.07±0.10 2.84±0.24 *0.35*

SHDLP 9.41±0.15 9.07±0.30 *0.35*

APOA1 132.5±1.6 124.2±3.8 ***0.034***

20 weeks

LDLP 1130±20 1100±36 *0.53*

LLDLP 305.6±9.8 280.5±18.2 *0.29*

MLDLP 241.2±16.5 228.8±43.2 *0.77*

SLDLP 583.6±20.4 590.3±54.0 *0.90*

APOB 63.3±1.1 62.3±1.9 *0.71*

HDLP 16.8±0.2 16.4±0.5 *0.37*

LHDLP 5.40±0.10 4.90±0.23 *0.051*

MHDLP 2.14±0.08 2.64±0.21 ***0.017***

SHDLP 9.29±0.13 8.85±0.32 *0.17*

APOA1 136.7±1.8 132.7±4.3 *0.37*

30 weeks

LDLP 1360±26 1269±41 *0.069*

LLDLP 391.2±10.9 390.2±22.4 *0.97*

MLDLP 164.0±14.2 205.8±39.7 *0.26*

SLDLP 804.8±24.2 673.4±49.6 ***0.030***

APOB 77.3±1.4 73.9±2.5 *0.32*

HDLP 17.3±0.2 17.3±0.4 *0.97*

LHDLP 5.37±0.11 4.94±0.24 *0.11*

MHDLP 1.65±0.07 1.90±0.16 *0.15*

SHDLP 10.3±0.1 10.4±0.3 *0.53*

APOA1 140.9±1.6 137.6±3.7 *0.40*

*NBW-Normal birth weight, LBW-Low birth weight

Values represent means ± SEM, adjusted for maternal BMI. Bold *P* values are significant.

Table S4. Relative amounts of proteins in APOB depleted plasma during gestation

Protein Enrollment 20 wks 30 wks *P value**

Albumin (ALBU) 16.42±0.24 ^a^ 16.59±0.24 ^a^ 10.40±0.24 ^b^ <*0.0001*

Complement factor 3 (CO3) 8.31±0.08 ^a^ 7.23±0.08 ^b^ 9.26±0.08 ^c^ <*0.0001*

Apolipoprotein A-I (APOA1) 6.39±0.09 ^a^ 6.95±0.09 ^b^ 6.58±0.09 ^a^ <*0.0001*

Complement factor 4A (CO4A) 4.08±0.05 ^a^ 4.62±0.05 ^b^ 4.85±0.05 ^c^ <*0.0001*

Complement factor 4B (CO4B) 3.96±0.07 ^a^ 4.62±0.07 ^b^ 4.85±0.07 ^b^ <*0.0001*

Ig gamma-4 chain C region (IGHG1) 3.51±0.05 ^a^ 2.99±0.05 ^b^ 2.47±0.05 ^c^ <*0.0001*

Apolipoprotein A-II (APOA2) 3.45±0.07 ^a^ 3.23±0.07 ^a^ 1.60±0.07 ^b^ <*0.0001*

Ig gamma-3 chain C region (IGHG3) 2.70±0.04 ^a^ 2.03±0.04 ^b^ 1.72±0.07 ^c^ <*0.0001*

Fibrinogen Gamma Chain (FIBG) 2.55±0.04 ^a^ 1.74±0.04 ^b^ 2.00±0.04 ^c^ <*0.0001*

Vitamin D Binding Protein (VTDB) 2.43±0.07 ^a^ 3.46±0.07 ^b^ 2.76±0.07 ^c^ <*0.0001*

Alpha-2-HS-Glycoprotein (FETUA) 2.06±0.04 ^a^ 1.80±0.04 ^b^ 1.94±0.04 ^a,b^ *0.0002*

Complement Factor H (CFAH) 2.03±0.05 2.02±0.05 2.13±0.05 *0.26 ^Ψ^*

Ceruloplasmin (CERU) 2.02±0.05 ^a^ 1.57±0.05 ^b^ 2.78±0.05 ^c^ <*0.0001*

Complement Factor B (CFAB) 1.94±0.04 ^a^ 2.41±0.04 ^b^ 2.49±0.04 ^b^ <*0.0001*

Fibrinogen Beta Chain (FIBB) 1.88±0.03 ^a^ 1.81±0.03 ^a^ 1.57±0.03 ^b^ <*0.0001*

Kininogen 1 (KNG1) 1.80±0.03 ^a^ 1.39±0.03 ^b^ 1.78±0.03 ^a^ <*0.0001*

Ig gamma-4 chain C region (IGHG4) 1.68±0.07 1.46±0.07 1.44±0.07 *0.005 ^Ψ^*

Haptoglobin (HPT) 1.63±0.04 ^a^ 1.27±0.04 ^b^ 1.70±0.04 ^a^ <*0.0001*

Inter-Alpha-Trypsin Inhibitor Heavy Chain 4 (ITIH4) 1.62±0.04 ^a^ 1.45±0.04 ^b^ 1.84±0.04 ^c^ <*0.0001*

Alpha-1 antitrypsin (A1AT) 1.56±0.04 ^a^ 1.81±0.04 ^b^ 1.31±0.04 ^c^ <*0.0001*

Ig kappa chain C region (IGKC) 1.43±0.03 ^a^ 1.65±0.03 ^b^ 1.26±0.03 ^c^ <*0.0001*

Ig gamma-2 chain C region (IGHG2) 1.42±0.03 ^a^ 1.16±0.03 ^b^ 1.36±0.03 ^a^ <*0.0001*

Fibrinogen Alpha Chain (FIBA) 1.41±0.04 ^a^ 1.73±0.04 ^b^ 1.96±0.04 ^c^ <*0.0001*

Haptoglobin-related protein (HPTR) 1.23±0.04 ^a^ 0.83±0.04 ^b^ 1.32±0.04 ^a^ <*0.0001*

Plasminogen (PLMN) 1.15±0.03 ^a^ 1.49±0.03 ^b^ 1.33±0.03 ^c^ <*0.0001*

Clusterin (CLUS) 1.04±0.02 ^a^ 1.17±0.02 ^b^ 1.26±0.02 ^c^ <*0.0001*

Ig mu chain C region (IGHM) 0.99±0.03 ^a^ 0.87±0.03 ^b^ 0.85±0.03 ^b^ <*0.0001*

Immunoglobulin Lambda-Like Polypeptide 5 (IGLL5) 0.94±0.03 1.04±0.03 ND^Φ^ *0.04 ^Ψ^*

Apolipoprotein A-IV (APOA4) 0.83±0.03 ^a^ 0.90±0.02 ^a^ 1.46±0.03 ^b^ <*0.0001*

Gelsolin (GELS) 0.80±0.02 ^a^ 0.93±0.02 ^b^ 1.00±0.02 ^b^ <*0.0001*

Inter-Alpha-Trypsin inhibitor Heavy Chain H2 (ITIH2) 0.80±0.02 ^a^ 0.92±0.02 ^b^ 1.17±0.02 ^c^ <*0.0001*

Paraoxonase 1 (PON1) 0.75±0.02 ^a^ 0.73±0.02 ^a^ 0.88±0.02 ^b^ <*0.0001*

Inter-Alpha-Trypsin inhibitor Heavy Chain H1 (ITIH1) 0.75±0.02 ^a^ 0.73±0.02 ^a^ 1.06±0.02 ^b^ <*0.0001*

Vitronectin (VTNC) 0.75±0.02 ^a^ 0.91±0.02 ^b^ 0.96±0.02 ^b^ <*0.0001*

Hemopexin (HEMO) 0.71±0.03 ^a^ 0.76±0.03 ^a^ 0.90±0.03 ^b^ <*0.0001*

Apolipoprotein E (APOE) 0.70±0.03 ^a^ 0.85±0.03 ^b^ 1.23±0.03 ^c^ <*0.0001*

Histidine-Rich Glycoprotein (HRG) 0.68±0.01 ^a^ 0.52±0.01 ^b^ 0.38±0.01 ^c^ <*0.0001*

Complement Factor 9 (CO9) 0.67±0.02 ^a^ 0.78±0.02 ^b^ 0.86±0.02 ^c^ <*0.0001*

Ig alpha-1 chain C region (IGHA1) 0.60±0.02 ^a^ 0.43±0.02 ^b^ 0.79±0.02 ^c^ <*0.0001*

Prothrombin (THRB) 0.53±0.02 ^a^ 0.68±0.02 ^b^ 0.73±0.02 ^b^ <*0.0001*

Alpha 1B-Glycoprotein (A1BG) 0.51±0.02 ^a^ 0.56±0.02 ^a^ 0.67±0.02 ^b^ <*0.0001*

Apolipoprotein H; Beta-2-Glycoprotein (APOH) 0.46±0.02 ^a^ 0.52±0.02 ^b^ 0.33±0.02 ^c^ <*0.0001*

Alpha-1-microglobulin/bikunin precursor (AMBP) 0.46±0.01 ^a^ 0.48±0.02 ^a^ 0.61±0.02 ^b^ <*0.0001*

Alpha 2-MacroGlobin (A2MG) 0.44±0.02 ^a^ 0.24±0.02 ^b^ 0.43±0.02 ^a^ <*0.0001*

Serotransferrin (TRFE) 0.39±0.02 ^a^ 0.35±0.02 ^a^ 0.50±0.02 ^b^ <*0.0001*

Heparin Cofactor 2 (HEP2) 0.35±0.01 ^a^ 0.35±0.01 ^a^ 0.52±0.01 ^b^ <*0.0001*

Complement Factor 5 (CO5) 0.31±0.01 ^a^ 0.31±0.01 ^a^ 0.41±0.02 ^b^ <*0.0001*

Apolipoprotein C-III (APOC3) 0.31±0.01 ^a^ 0.36±0.01 ^b^ 0.38±0.01 ^b^ <*0.0001*

Complement Factor I (CFAI) 0.30±0.02 ^a^ 0.39±0.02 ^b^ 0.44±0.02 ^b^ <*0.0001*

Pigment Epithelium-Derived Factor (PEDF) 0.30±0.01 0.31±0.01 0.33±0.01 *0.20 ^Ψ^*

Angiotensinogen (ANGT) 0.29±0.02 ^a^ 0.31±0.02 ^a^ 0.73±0.02 ^b^ <*0.0001*

Transthyretin (TTHY) 0.27±0.02 ^a^ 0.14±0.02 ^b^ 0.31±0.02 ^a^ <*0.0001*

Complement Factor 6 (CO6) 0.26±0.02 ^a^ 0.39±0.02 ^b^ 0.38±0.02 ^b^ <*0.0001*

Complement Factor 7 (CO7) 0.23±0.02 ^a^ 0.32±0.02 ^b^ 0.45±0.02 ^c^ <*0.0001*

Insulin-like Growth Factor-Binding Protein Complex (ALS) 0.23±0.02 ^a^ 0.09±0.02 ^b^ 0.27±0.02 ^a^ <*0.0001*

Apolipoprotein C-I (APOC1) 0.23±0.01 ^a^ 0.17±0.01 ^b^ 0.16±0.01 ^b^ <*0.0001*

Complement C1R Subcomponent (C1R) 0.21±0.01 ^a^ 0.28±0.01 ^b^ 0.31±0.01 ^b^ <*0.0001*

Complement C1S Subcomponent (C1S) 0.19±0.01 ^a^ 0.25±0.01 ^b^ 0.32±0.01 ^c^ <*0.0001*

Apolipoprotein L1 (APOL1) 0.18±0.01 ^a^ 0.34±0.01 ^b^ 0.53±0.01 ^c^ <*0.0001*

C4B-Binding Protein Alpha Chain (C4BPA) 0.16±0.01 ^a^ 0.26±0.01 ^b^ 0.13±0.01 ^a^ <*0.0001*

**P* values are those with significant False Discovery Rates.

^Φ^ND-Not detected.

Values represent unadjusted means ± SEM.

Table S4 (cont). Relative amounts of proteins in APOB depleted plasma during gestation

Protein Enrollment 20 wks 30 wks *P value**

Immunoglobin J Chain (IGJ) 0.16±0.01 ND ^Φ^ 0.18±0.01 *0.22 ^Ψ^*

Pregnancy Zone Protein (PZP) 0.15±0.03 ^a^ 0.39±0.03 ^b^ 0.59±0.03 ^c^ <*0.0001*

Retinol Binding Protein 4 (RET4) 0.12±0.01 ^a^ 0.14±0.01 ^b^ 0.10±0.01 ^a^ <*0.0001*

Afamin (AFAM) 0.11±0.01 ^a^ ND 0.18±0.02 ^b^ *0.0005*

Serum Amyloid A 4 (SAA4) 0.11±0.01 ^a^ 0.13±0.01 ^b^ 0.18±0.01 ^c^ <*0.0001*

Fetuin-B (FETUB) 0.10±0.01 ^a^ 0.17±0.01 ^b^ 0.11±0.01 ^a^ <*0.0001*

Kallistatin (KAIN) 0.09±0.01 0.06±0.01 0.06±0.01 *0.03 ^Ψ^*

N-Acetylmuramoyl-L-alanine amidase (PGRP2) 0.08±0.01 ^a^ 0.12±0.01 ^b^ 0.06±0.01 ^a^ *0.0002*

Coagulation factor XII (FA12) 0.05±0.01 ^a^ 0.08±0.01 ^b^ 0.12±0.01 ^c^ <*0.0001*

Ig Lambda-2 Chain C Regions (LAC2) ND 1.15±0.02 ^a^ 0.83±0.02 ^b^ <*0.0001*

Apolipoprotein C-II (APOC2) ND 0.22±0.01 ^a^ 0.49±0.01 ^b^ <*0.0001*

Complement Factor H-related protein 1 (FHR1) ND 0.16±0.01 0.11±0.01 *0.01 ^Ψ^*

Carboxypeptidase N subunit 2 (CPN2) ND 0.15±0.01 0.17±0.01 *0.35 ^Ψ^*

Pregnancy Specific Glycoprotein 1 (PSG1) ND 0.12±0.01 ^a^ 0.43±0.01 ^b^ <*0.0001*

Immunoglobin Kappa Variable 3-20 (KV320) ND 0.10±0.01 0.10±0.01 *0.89 ^Ψ^*

Alpha-2-antiplasmin (A2AP) ND 0.09±0.01 ^a^ 0.16±0.01 ^b^ <*0.0001*

Complement Factor 8B (CO8B) ND 0.09±0.01 0.10±0.01 *0.23 ^Ψ^*

Proteins detectable at only one time point

Immunoglobin Kappa variable (IGK) 1.37±0.04 ND ND

Ig Lambda-2 chain C regions (IGLC2) 0.80±0.03 ND ND

Tetranectin (TETN) 0.06±0.01 ND ND

Ig Heavy Chain V-III (HV311) ND 0.14±0.01 ND

Complement C1q Subcomponent Subunit C (C1QC) ND 0.11±0.01 ND

Complement Factor 2 (CO2) ND 0.06±0.01 ND

Pregnancy Specific Glycoprotein 4 (PSG4) ND ND 0.25±0.01

Sex Hormone-Binding Globulin (SHBG) ND ND 0.16±0.01

Pregnancy Specific Glycoprotein 3 (PSG3) ND ND 0.16±0.02

Inter-Alpha-Trypsin inhibitor Heavy Chain H3 (ITIH3) ND ND 0.12±0.00

Complement Factor H-related Protein 2 (FHR2) ND ND 0.01±0.01

Carboxypeptidase N Catalytic Chain (CBPN) ND ND 0.09±0.01

Chorionic somatomammotropin hormone 1 (CSH1) ND ND 0.09±0.01

Fibulin 1 (FLBN1) ND ND 0.08±0.01

Complement C1q Subcomponent Subunit B (C1QB) ND ND 0.06±0.01

Ficolin-3 (FCN3) ND ND 0.06±0.01

Values represent adjusted means ± SEM. Values with different superscripts are significantly different at p<0.05 after Bonferroni adjustment, as appropriate. For pairwise comparisons among 3 values, p<0.017 (=0.05/3 tests) is considered significant.

*^Ψ^* Overall *P* values not significant at Bonferroni-corrected p<0.00064 (=0.05/82 tests), and pairwise differences among values not presented.

**P* value for differences among time points from linear mixed models adjusted for maternal pre-pregnancy BMI, infant birth weight

and gestational age at birth.

^Φ^ND-Not detected.

Supplemental Table 5. GO Pathway Proteins

Metal Ion Complement Acute Inflam. Protease Lipid

Binding Activation Response Hemostasis Inhibition Metabolism

APOA4 C4BPA A1AT A1AT A1AT ANG1

APOE CLUS FETUA A2MG FETUA APOA1

CERU C1QB APOA2 APOA1 A2MG APOA2

C1R C1QC C4BPA APOH MABP APOA4

C1S C1R CLUS CLUS ANGT APOC1

FIBG C1S C1QB CO3 CO3 APOC2

HEMO CO3 C1QC FIBA CO4A APOC3

HRG CO4A C1R FIBB CO4B APOE

ITIH1 CO4B C1S FIBG HRG APOL1

KNG1 CFAB CO3 HRG ITIH1 APOM

PON1 CFAH CO4A KNG1 ITIH2 APOH

PGRP2 IGHA1 CO4B PLMN ITIH4 CLUS

THRB IGHG1 CFAB THRB KAIN PONQ

TRFE IGHG2 CFAH TRFE KNG1 TTHY

IGHG3 FIBA PZP VTDB

IGHG4 HPT

IGKC IGHA1

LAC2 IGHG1

IGHM IGHG2

IGLL5 IGHG3

IGHG4

IGKC

LAC2

IGHM

IGLL5

IGIH4

THRB

SAA4

Supplemental Table 6. Top proteins in HDL of pregnant women

Cincinnati Gambia

ALBU ALBU

APOA1 CO3

CO3 APOA1

IGHG1 CO4B

**FIBA*** **CO4A**

CO4B VTDB

**HPT** **APOA2**

VTDB IGHG1

**FIBG** **CFAB**

**FIBB** **IGHG3**

*Bold proteins are different in the two

cohorts of women in mid-gestation.
